# Special Issue “Human and Animal Monocytes and Macrophages in Homeostasis and Disease: 5th Edition”

**DOI:** 10.3390/ijms26178180

**Published:** 2025-08-23

**Authors:** Malgorzata Kloc, Jacek Z. Kubiak

**Affiliations:** 1 The Houston Methodist Research Institute, Houston, TX 77030, USA; 2 Department of Surgery, The Houston Methodist Hospital, Houston, TX 77030, USA; 3 Department of Genetics, MD Anderson Cancer Center, The University of Texas, Houston, TX 77030, USA; 4 Dynamics and Mechanics of Epithelia Group, Institute of Genetics and Development of Rennes (IGDR), National Centre for Scientific Research (CNRS), Faculty of Medicine, University of Rennes, UMR 6290, 35043 Rennes, France; jacek.kubiak@univ-rennes.fr; 5 Laboratory of Molecular Oncology and Innovative Therapies, Military Institute of Medicine-National Research Institute, Szaserow 128, 04-141 Warszawa, Poland

The articles published in this Special Issue cover various topics, as detailed below.

The first study in this Special Issue examined the impact of the β-adrenergic receptor (β-AR)-blocking drug propranolol on macrophage differentiation and polarization, specifically in promoting inflammatory and anti-inflammatory phenotypes. Propranolol upregulated the expression of the oxidative stress regulators NRF2, heme oxygenase-1, and NQO1, indicating its therapeutic potential as a novel anti-inflammatory and immunomodulating compound for numerous inflammatory diseases.

In the second article featured in this Special Issue, the authors assessed the effect of fever-range hyperthermia (FRH) on macrophages, which are highly susceptible to heat. In the study, macrophages exposed to FRH were polarized toward an M2 phenotype, increasing levels of CD163, IL-10, Arg-1, COX-2, TNF-α, and TLR-4 and reducing antimicrobial molecules ROS and nitric oxide (NO); this indicates the therapeutic potential of FHR. The third study examined the anti-inflammatory effects of Tegoprazan (TEGO) on bone marrow-derived macrophages, demonstrating that TEGO decreased nitric oxide (NO) production in BMMs. It also reduced the level of pro-inflammatory cytokines and increased the expression of anti-inflammatory cytokines, suggesting its capacity to function as an anti-inflammatory therapeutic. Macrophage-targeted therapy is additionally discussed in the fourth article, specifically as a treatment for acute respiratory distress syndrome (ARDS)—a lung condition characterized by hyperinflammation and fibrosis. The authors discerned a relationship between the monocyte-derived enzyme adenosine deaminase 2 (ADA2) and SARS-CoV-2-induced ARDS, as well as a link between ADA2 and the chemokine CXCL10 and its receptor CXCR3. By examining published datasets of spatial transcriptomics and single-cell RNAseq, the authors determined that ADA2 is highly expressed by inflammatory CD14+CD16+ monocytes in lungs affected by ARDS.

The fifth article analyzes the impact of growth differentiation factor 15 (GDF-15), a multifunctional cytokine from the transforming growth factor-beta (TGF-β) superfamily. Macrophages produce high levels of GDF-15 during stress, which can result in fibrogenesis and angiogenesis. These cells also respond to GDF-15 by switching to a tolerogenic phenotype. The authors discuss existing and novel GDF-15-based therapies, such as GDF-15 analogs/mimetics and GDF-15-targeting monoclonal antibodies. In the next article, the role of monocytes and macrophages in kidney homeostasis, disease, and different kidney compartments is investigated.

The final paper reviews invertebrate immunity, a highly understudied subject. It describes the cellular and molecular aspects of invertebrate immunity, including the epigenetic foundation of innate memory, the transgenerational inheritance of immunity, genetic immunity against invading transposons, the mechanisms of self-recognition, natural transplantation, and germ/somatic cell parasitism.

Most of the articles published in this Special Issue explore therapeutic approaches that target macrophages. We additionally seek the submission of papers that focus on the basic science aspects of monocyte/macrophages. These include regulatory macrophages [1], metabolically activated macrophages [2], lipid-associated macrophages [3], memory macrophages [4], innate immune memory [5], and the role of macrophage fusion in the creation of homo- and heterotypic syncytia [6], not only in humans and mammalian models but in various vertebrates and invertebrate species as well [7].
